# Coping Power-Rural: A Pilot Study of the Two-Tiered Preventive Intervention Focused on Addressing Transdiagnostic Needs in Rural Schools

**DOI:** 10.1007/s12310-026-09873-3

**Published:** 2026-04-21

**Authors:** Catherine P. Bradshaw, Courtney G. Newman, Katelyn Birchfield, Jamie DeCoster, Paloma Pérez, Candra Skrzypek, Jacqueline Hersh, Lydia Anne Beahm, Amanda Nguyen

**Affiliations:** 1 University of Virginia, Charlottesville, USA; 2 University of North Florida, Jacksonville, USA; 3 Appalachian State University, Boone, USA; 4 Clemson University, Clemson, USA

**Keywords:** Social and emotional learning, Tiered interventions, Behavioral support, Rural programming

## Abstract

This paper reports initial findings from a pilot study of the Coping Power-*Rural* program with upper elementary and middle school students, which was created to meet the specific needs of rural populations. Coping Power-*Rural* included adapted content to ensure it was relevant and accessible to students and teachers in rural schools. The program followed a two-tiered delivery format, incorporating both universal classroom sessions as well as targeted group sessions. The program also promoted skill generalization across contexts (e.g., school and home settings) through a flexible, technology-enhanced caregiver component, which provided caregivers with remote access to resources and supports. Building on prior feasibility and acceptability work (Nguyen et al. in Sch Ment Health 16:776–792, 2024. 10.1007/s12310-024-09632-2), this pilot study reports pre-post contrast testing of the Coping Power-*Rural* program in six rural schools across three states, involving 185 students (grades 4–7; 55.1% male; 43.2% Black; 7.6% Latine) and 48 school-based staff. The pre-post pilot outcomes indicated significant improvements in student conduct problems, aggressive/disruptive behavior, prosocial behavior, emotion regulation problems, internalizing problems, family stability, and family involvement from pretest to posttest (*p* < 0.05; Cohen’s *d*s ranged from 0.20 to 0.48). There were also significant pre-post improvements for youth who participated in the targeted group on several teacher-reported outcomes (e.g., greater reductions in aggressive/disruptive behavior, emotion regulation problems, internalizing problems). These pre-post pilot findings provide promising evidence regarding outcomes for students based on exposure to the classroom Coping Power-*Rural* sessions, as well as for students in the targeted group.

**Supplementary Information:**

The online version contains supplementary material available at 10.1007/s12310-026-09873-3.

## Introduction

Rural communities face unique challenges that necessitate increased mental health supports. Caregivers (i.e., parents and guardians) and educators of children living in rural areas report concern for student mental health needs (e.g., internalizing and externalizing behaviors; Garbacz et al., 2022). Research suggests that children living in rural areas have elevated behavior challenges (e.g., oppositional defiant disorder) and internalizing symptomology (e.g., depression, anxiety) compared to children living in urban areas (Bitsko et al., 2022). These difference may be due to structural inequities faced by those living in rural communities, including reduced education for parents and caregivers, a lack of accessible mental health services, or financial strain (Bitsko et al., 2022). For example, rural populations face substantial mental health provider shortages with fewer specialty mental health providers serving their communities (Andrilla et al., 2018). In addition, rural residents may find it difficult to access services due to limited public transportation and the need to drive long distances to reach providers (Blackstock et al., 2018).

School systems throughout the United States play a pivotal role in providing youth in rural school districts access to mental health services (Blackstock et al., 2018). This is particularly crucial in areas where mental health provider shortages persist. However, compared to students in suburban and urban localities, students in rural schools have significantly less access to mental health services at school, including mental health assessment and evidence-based interventions (Shelton & Owens, 2021). Barriers to providing services in rural schools include inadequate access to qualified providers and inadequate funding (Graves et al., 2023; Shelton & Owens, 2021). Additional challenges to services may include a lack of shared understanding about mental health, limited caregiver engagement or lack of support for their child accessing services (e.g., stigma and negative attitudes towards mental health care), and reduced mental health literacy of teachers and caregivers (Blackstock et al., 2018; Garbacz et al., 2022; van Vulpen et al., 2018). Yet many caregivers believe that schools should help address youths’ mental health concerns. In a survey of over 600 rural caregivers, the majority were supportive of school involvement and of curriculum-based social-emotional lessons being implemented in classrooms (van Vulpen et al., 2018). Despite caregiver buy-in, common barriers to mental health supports and interventions include their cost-prohibitive and time- and resource-intensive nature.

The Coping Power*-Rural* program was adapted from the evidence-based Coping Power program (Lochman and Wells 2002a) to overcome some of these challenges and fit rural schools (Nguyen et al., 2024). Building on this strong research base, Coping Power-*Rural* employs a two-tiered approach, which includes a universal, classroom-wide program (Tier 1), coupled with a targeted group program (Tier 2) for students identified through a screening process. This paper presents findings from a pilot evaluation of the program with rural schools and students in grades 4 through 7, using a pre-post design. Coping Power (Lochman and Wells 2002a) is a rigorously tested prevention intervention program delivered in schools. The program was originally designed as a targeted intervention program for upper elementary students who display high levels of aggressive behavior; it is multifaceted and includes student, caregiver, and teacher components delivered by trained clinicians. Based on a contextual social-cognitive developmental model (Lochman and Wells 2002a), the program targets social-cognitive and self-regulation processes in youth. At the same time, family processes and practices are addressed through a caregiver component, and classroom-based support is provided for teachers (i.e., to promote school bonding, study and homework organizational skills, and academic success). The original Coping Power program has demonstrated numerous positive outcomes, including reducing delinquency, aggression, and substance misuse and improving self-regulation, concentration, on-task behavior, and academic achievement (Lochman et al. 2013; Lochman and Wells 2002a, 2002b, 2003; for a review, see Lochman & Bradshaw, in press).

The original program has since been adapted for adolescents with similar positive effects (Bradshaw et al., 2017, 2025). This adaptation utilized the Early Adolescent Coping Power (EACP) program, which was developmentally and contextually adapted to better fit the needs of middle school youth (i.e., grades 6–8) and schools (Bradshaw et al., 2017, 2025). While this version of the program retains the multicomponent structure of Coping Power (i.e., student, caregiver, and teacher components), it includes fewer student sessions, is designed to be delivered over a single school year, and incorporates content more applicable to upper elementary and middle school students, such as bullying (Bradshaw et al., 2025). Coping Power-*Rural*, which is the focus of the current study, was adapted from EACP to fit the context of rural schools. Further adaptations included delivering the program as a two-tiered transdiagnostic intervention, reducing the length of the program to 12 weeks, updating content to be contextually relevant, and using technology-assisted approaches to increase reach and engagement (see Nguyen et al., 2024 for additional details on the adaptation process, along with acceptability and feasibility findings).

While Coping Power was originally designed to reduce externalizing symptoms, its cognitive behavioral approach targets underlying processes of both internalizing and externalizing problems (e.g., improving emotional regulation and social problem-solving skills; Clifford et al., 2020). Thus, Coping Power-*Rural* offers utility as a transdiagnostic intervention, or one that simultaneously addresses multiple, often co-occurring problems (Hersh et al., 2016). For example, Mindfulness-Enhanced Coping Power has been shown to decrease suicidal thoughts and behaviors in youth over time (McDaniel et al., 2025). By maximizing the effects of interventions across externalizing and internalizing disorders, transdiagnostic interventions reduce the burden on schools by reducing the need to implement multiple programs with different targets (Clifford et al., 2020). This may be especially useful in rural contexts, where schools often face resource constraints and thus do not have the time or staffing to implement multiple programs simultaneously for various student groups. Coping Power-*Rural* was developed as a contextually relevant transdiagnostic program (Nguyen et al., 2024).

### Overview of Current Study

As described above, Coping Power-*Rural* (CP-R) was created to meet the specific needs of rural students and schools. This version of the program was adapted through a community-based participatory research approach to ensure it was relevant and accessible to students and teachers in rural schools (for a review of the adaptation process, and feasibility and acceptability data, see Nguyen et al., 2024). One of the key features of CP-R is its two-tiered delivery format, which includes universal classroom sessions as well as targeted group-based sessions for students identified through a screening process as being at risk and in need of more intensive supports. CP-R also incorporates a flexible, technology-enhanced caregiver component; this feature makes it easier for caregivers to participate in the program, receive information about the content of the sessions, and implement suggested strategies each week to reinforce student learning and encourage generalization across contexts. The program’s integration of coaching for school staff ensures that educators receive ongoing support and guidance, helping them to effectively apply the program’s principles in their schools. Moreover, the training was designed to be “light touch”, limiting the burden on implementers while anticipating skill diffusion through the faculty and staff through the electronically shared program materials. In this way, all adults in the school have the opportunity to become familiar with the intervention and its key topics, regardless of whether they received formal CP-R training.

The current pilot study was designed to build on a prior feasibility and acceptability study of the adapted program with a different sample (see Nguyen et al., 2024); the prior results indicated high acceptability and social validity ratings (Nguyen et al., 2024). Moreover, those findings indicated that technology-based enhancements, including a user-friendly website and video resources, supported implementation and sustainability in the participating rural and remote schools. Consistent with the iterative intervention development process (Kistin & Silverstein, 2015), the current pilot study aimed to evaluate the program using a pre-post design with a different sample of 185 4th through 7th graders. The first aim examined pre-post differences on teacher ratings of student behaviors, from baseline to the end of the school-based intervention. The second aim examined changes for youth who also participated in the targeted group based on teacher-reported outcomes. The third aim examined educator-reported changes; specifically, teacher feelings related to school climate, burnout, and overall wellbeing of students and staff. Together, these three aims were intended to build on the prior feasibility and acceptability research to provide insight on, and estimate the effect size of, potential pre-post changes for students’ exposure to the classroom CP-R lessons, and for youth exposure to the CP-R targeted group sessions. Additionally, this study sought to capture changes at the educator-level for those working with the students in these grades (Kistin & Silverstein, 2015).

## Method

### Participants

Data for this pilot study were collected during the 2023–2024 school year and included 48 school staff (i.e., teachers, counselors, psychologists) and 185 students in grades 4–7 from six schools in five districts across three southeastern states (Virginia, North Carolina, and South Carolina). According to the National Center for Education Statistics classification system, four participating schools were categorized as rural fringe, one as rural distant, and one as suburban small. These classifications reflect primarily rural contexts with varying proximity to urban centers. The student participants (*n* = 185) were 43.8% were female, 55.1% male, 43.2% Black, and 7.6% Latine in grades 4–7. Of the school staff who consented into the project, 20.8% fulfilled roles other than teaching (e.g., school psychologists, administrators, and family liaisons). Overall, school staff were predominantly White (*n* = 39, 81.3%) and female (*n* = 45, 93.8%). See Table 1 for additional student and staff participant characteristics (and Supplemental Table A).

**Table 1:** Participant characteristics

Characteristic	Count (percent) or mean (standard deviation)			
	Pretest		Posttest	
	Student	Teacher	Student	Teacher
*Grade level*				
4th Grade	59 (31.9%)	8 (16.7%)	62 (32.1%)	8 (16.3%)
5th Grade	45 (24.3%)	7 (14.6%)	54 (28.0%)	8 (16.3%)
6th Grade	39 (21.1%)	2 (4.2%)	36 (18.7%)	2 (4.1%)
7th Grade	40 (21.6%)	5 (10.4%)	40 (20.7%)	4 (8.2%)
Multiple grades	–	16 (33.4%)	–	21 (42.8%)
Did not teach	–	10 (20.8%)	–	6 (12.2%)
Unreported	2 (1.1%)	–	1 (0.5%)	–
*Race/ethnicity*				
Black/African American	80 (43.2%)	9 (18.8%)	79 (40.9%)	8 (16.3%)
Hispanic/Latine	14 (7.6%)	–	10 (5.2%)	–
White	61 (33.0%)	39 (81.3%)	64 (33.2%)	39 (79.6%)
Multiracial or Other	26 (13.5%)	0 (0.0%)	34 (17.6%)	2 (4.0%)
Unreported	4 (2.2%)	–	6 (3.1%)	–
*Sex*				
Female	81 (43.8%)	45 (93.8%)	86 (44.6%)	45 (91.8%)
Male	102 (55.1%)	3 (6.3%)	101 (52.3%)	4 (8.2%)
Self-Identify	0 (0.0%)	–	5 (2.6%)	–
Unreported	2 (1.1%)	–	1 (0.5%)	–
Age (M ± SD)	10.96 ± 1.45	–	11.09 ± 1.38	–
*Support received*				
Tier 1	151 (81.6%)	–	161 (83.4%)	–
Tier 1 and Tier 2	34 (18.4%)	–	32 (16.6%)	–
*Received training*				
No	–	32 (66.7%)	–	35 (71.4%)
Yes	–	16 (33.3%)	–	14 (28.6%)
*Rated students*				
No	–	28 (58.3%)	–	29 (59.2%)
Yes	–	20 (41.7%)	–	20 (40.8%)

### Recruitment

#### Schools

School recruitment utilized a two-pronged approach, including leveraging prior connections and partnerships as well as eliciting new partnerships through email outreach. In total, 19 school divisions were contacted after expressing interest in project participation. Six schools, including five elementary schools and one middle school, completed the commitment and consent process to engage in the project. Although the sample of schools was unbalanced across elementary vs. middle, the number of students was relatively balanced across grade level (i.e., grades 4–7, see Table 1).

#### School Employees

First, schools received a district-level letter of approval for their participation in the project. The research team then worked with school-based administrative and counseling teams to recruit teachers, school counselors, and school psychologists. All participants provided written consent for their participation, both for the completion of surveys and student behavioral ratings. All of the schools’ counselors (100%) who were approached about the project provided their consent to implement the program, and similarly 100% of all classroom teachers provided their consent to complete the surveys and behavior rating scales of students where appropriate; 9 of the recruited teachers did not complete the follow-up surveys due to parental leave, departure from the school, death , or general loss to follow-up. As described below, in the majority of cases, the program implementation was completed by school counselors or psychologists given the limited bandwidth of classroom teachers.

#### Students

Participating school counselors and administrators used a flyer provided by the research team to disseminate information to caregivers. Flyers were sent home in student backpacks or through electronic newsletters. Caregivers were asked to consent via Qualtrics or to return the flyer with a signature indicating consent for their child to participate in the project or declining their students’ involvement in the research. Once the caregiver consent was obtained, students were asked to provide their assent. Study attrition occurred for only four students, all due to family relocations. No families revoked consent for child participation during the intervention or before the follow-up data collection, although four students were lost to follow-up, due to absence or relocation before post-test.

### Training and Implementation Coaching

#### Implementation Planning, Training, and Supports

The program implementation support process began with a school action planning meeting, which was led by the project-hired coach and attended by administrators, counselors, and potential implementers. The purpose of the action planning meeting was to establish a clear foundation for the program, address logistical elements such as the implementer training date, finalizing the implementation timeline, and determining the process for collecting student and staff consent and completing data collection. By involving key stakeholders early, the planning meeting aimed to set the stage for a collaborative and organized implementation. This session was led by the CP-R project manager and the school’s assigned project coach to ensure that school stakeholders had multiple points of contact within the research team. In total, there were four project coaches for this pilot study, all of whom are co-authors on this paper.

After this initial meeting with administration and key stakeholders, the research team held a required initial training session for all implementers. In all but one case, the school counselor and mental health support team (i.e., school psychologists) were the implementers of the program. The one middle school did opt to have the general education classroom teachers deliver the program, and therefore their classroom teachers were involved in planning meetings and trainings. Consistent with the intent for the program training to be “light-touch” for implementers, training was provided to this subset of implementers (*n* = 16, 33.3%) working with students in the specified participating grades; however, all participating educators (*n* = 48) received the weekly electronic program nudges and access to the electronic materials on the program. While training was required for all implementers of the program, other school staff and administrators were invited as optional attendees to further develop capacity. The training session was led by the project manager and the school’s coach and lasted between 60 and 90 min in duration. During the training, attendees (including targeted group and classroom implementers) were provided with a broad overview of the curriculum and themes, taught how to access materials via the website, provided with tips for their first weeks of implementation, and given an opportunity to plan their implementation and ask questions of the research team. One training session was completed for each school, although two schools in the same district opted to complete their training together, leading to a total of five initial trainings for the six schools.

To further increase educator and implementer knowledge of program content, all implementers and educators interacting with the students in the program received the weekly electronic infographic “nudges” via email. These materials provided an overview of program content and implementation tips for the general education classroom setting. All teachers, counselors, psychologists, and administrators, and other interested staff held accounts to access the online Coping Power-*Rural* website and program resources.

#### Coaching

Once the planning and training stages were completed, coaching sessions and implementation began. The Coping Power-*Rural* program includes three required coaching sessions with implementers at regular intervals to provide structured, ongoing support and to ensure fidelity of implementation. The coaching followed a structured coaching implementation protocol and was tracked by a set of logs and measures to document dosage and fidelity to the coaching model. The first coaching session occurred between the initial training session and the start of program implementation; it provided implementers with a base level of familiarity with CP-R content before delving deeper into implementation specifics. This first session focused on motivational interviewing to build buy-in among implementers and to plan for effective program integration into school routines. The two subsequent coaching sessions occurred after approximately the third and seventh lesson. In these two coaching sessions, the focus shifted to reviewing weekly logs submitted by implementers to discuss progress and address challenges (e.g., student behavioral concerns, scheduling conflicts). These sessions also provided an opportunity for continuous improvement and refinement in the coaching support and program implementation as needed (for additional detail on the coaching model, see Nguyen et al., 2024). In addition to the three required coaching sessions, coaches provided weekly updates and reminders to all implementers via email. Coaches were also available for on-demand coaching upon request to individual implementers. At the conclusion of the program, implementers were provided with tips and strategies to generalize the program practices and sustain them throughout the school year and beyond. Project coaches completed an electronic log of the contacts with the staff at their schools (e.g., emails, Zoom and phone sessions), as well as ratings of fidelity and implementer engagement during the coaching sessions. The details regarding content and completion rates of these structured coaching sessions were documented in the coaching fidelity checklist and are summarized in Table 2.

**Table 2:** Program implementation data

Source	Count (%)	Total time spent in minutes (%)	Mean time spent in minutes (Standard Deviation)
*A. coaching data collected through the coach logs* ^a^			
Modality			
Email	88 (72.1%)	815 (27.5%)	9.3 (8.8)
In-person meeting	9 (7.4%)	1295 (43.8%)	143.9 (104.1)
Online meeting	18 (14.6%)	760 (25.7%)	42.4 (17.2)
Phone call	2 (1.6%)	65 (2.25)	32.5 (10.6)
Text	5 (4.1%)	25 (0.8%)	5.0 (0)
Purpose			
Ad Hoc Coaching	28 (23.0%)	515 (17.4%)	18.4 (31.3)
Coaching Session 1	9 (7.4%)	330 (11.1%)	36.7 (24.0)
Coaching Session 2	4 (3.3%)	125 (4.2%)	31.3 (22.5)
Coaching Session 3	3 (2.5%)	120 (4.1%)	40.0 (17.3)
Initial Training†	5 (4.1%)	300 (10.1%)	60.0 (37.2)
Mterials Support	16 (13.1%)	260 (8.8%)	16.3 (16.7)
Outreach and Planning	2 (1.6%)	90 (3.0%)	45 (21.2)
Research Rollout	5 (4.1%)	640 (21.6%)	128 (145.3)
Research Wrap-Up	2 (1.6%)	255 (8.6%)	127.5 (159.1)
Weekly Email	48 (39.3%)	325 (11.0%)	6.8 (2.8)
Total	122 (100.0%)	2960 (100.0%)	24.3 (45.9)

	Number (%) endorsed		
	Time 1	Time 2	Time 3
*B. Program implementation data*			
Initial engagement/rapport building	5 of 7 (71.4%)	7 of 7 (100%)	7 of 7 (100%)
Complete motivational interview	6 of 7 (85.7%)	7 of 7 (100%)	7 of 7 (100%)
Review teacher-level action planning guide	5 of 7 (71.4%)	7 of 7 (100%)	7 of 7 (100%)
Demonstrate access of Google Drive	7 of 7 (100%)	7 of 7 (100%)	7 of 7 (100%)
Complete the weekly fidelity log	6 of 7 (85.7%)	3 of 7 (42.9%)	5 of 5 (100%)
Reflect and schedule time to meet again (for sessions 1 and 2)	6 of 7 (85.7%)	5 of 7 (71.4%)	7 of 7 (100%)
The implementer appeared comfortable and willing to participate	7 of 7 (100%)	3 of 7 (42.9%)	5 of 5 (100%)
The implementer appeared motivated and excited for implementation	7 of 7 (100%)	7 of 7 (100%)	7 of 7 (100%)

	Count (%) or mean (Standard Deviation)	
	Classroom	Targeted Group
*C. Program facilitation, attendance, and core program components delivered*		
Teacher present for lesson delivery		
Yes	81 (55.5%)	0 (0%)
Partially	9 (6.2%)	0 (0%)
No	56 (38.4%)	71 (100%)
Warm up performed		
Yes	145 (90.1%)	60 (87.0%)
Partially	14 (8.7%)	7 (10.1%)
No	2 (1.2%)	2 (2.9%)
Skill introduction performed		
Yes	148 (89.7%)	67 (94.4%)
Partially	17 (10.3%)	0 (0%)
No	0 (0%)	4 (5.6%)
Skill practice performed		
Yes	137 (83.0%)	54 (76.1%)
Partially	28 (17.0%)	12 (16.9%)
No	0 (0%)	5 (7.0%)
Wrap up performed		
Yes	134 (81.2%)	52 (73.2%)
Partially	25 (15.2%)	13 (18.3%)
No	6 (3.6%)	6 (8.5%)
Staff comfortable		
Yes	142 (86.6%)	71 (100%)
Partially	22 (13.4%)	0 (0%)
No	0 (0%)	0 (0%)
Students comfortable		
Yes	150 (92.0%)	56 (78.9%)
Partially	13 (8.0%)	15 (21.1%)
No	0 (0%)	0 (0%)
Number of students	16.87 (4.67)	5.87 (2.31)
Percent of students participating	80.11 (23.02)	93.97 (11.35)

^a^Count refers to the number of times a coaching modality was utilized and its purpose. Total Time Spent refers to the amount of minute a coach spent engaging in a coaching modality. Mean Time Spent refers to the average time spent in specified coaching modalities and purposes

^†^Two schools within one of the districts trained together, for a total of 5 trainings for 6 schools

#### Implementation: Classroom

Once schools signed a commitment form, engaged in an action planning meeting, and implementers completed their program training and initial coaching session, implementation of the Coping Power-*Rural* curriculum began. The program was offered to all eligible classrooms (i.e., all 4th through 8th grade classrooms whose general education teacher had consented for project engagement). A total of 21 classrooms participated in the program, ranging from grades 4 through 7 and including 12 classrooms in Virginia, 6 classrooms in South Carolina, and 3 classrooms in North Carolina. In most cases, all students in the general education setting received the 12 universal classroom (Tier 1) lessons as a part of their regular programming during homeroom or counseling time. However, two schools did require students’ caregivers to provide separate consent for curriculum delivery. The children of any caregiver that did not consent to program exposure were sent to another classroom during the time of lesson implementation, where they engaged in alternative activities determined by the school. All other students received the 12 CP-R universal lessons in their general education classroom.

As noted above, the 12 universal lessons were in most cases provided by the school counselors or school psychologists, who also led the 12 targeted group sessions (i.e., Tier 2). The universal lessons were intended to be approximately 30–45 min, so they could fit within a classroom block. Whenever possible, general education teachers were asked to remain present in the classroom both to provide them with lesson exposure, so that they would have common language when discussing social-emotional challenges with their students. The session implementers submitted weekly fidelity data to track implementation.

#### Implementation: Targeted Group

In addition to the 12 universal lessons provided to all students at the Tier 1 level, targeted lessons were provided to a select group of students identified by classroom teachers as having a greater need for support in the areas of emotion regulation, self-management, social problem-solving, and coping skills. This targeted group component of the intervention consisted of an additional 12 lessons taught in a small pull-out group setting (range: 4 to 12 students). These targeted group sessions were held between each corresponding classroom lesson, so that the implementer could reinforce key concepts taught in the previous classroom session with targeted group. To promote generalization and concept reinforcement, the implementer would provide a brief review of key terms before engaging in various activities for practice and application, including debates, role-playing simulations, games, discussions, and other related activities. Like the universal lessons, these targeted group sessions were intended to last approximately 30–45 min and were led by the school counselor or psychologist. The targeted groups were conducted during typical classroom pull-out times, such as during homeroom or independent work time, to ensure that students engaging in the targeted group did not miss key academic programming, but also did not miss lunch, recess, or other important opportunities for social engagement.

#### Implementation: Caregiver Engagement

Lastly, generalization for students was promoted through the encouragement of caregiver engagement with a series of caregiver “nudges”. These weekly caregiver nudges were provided in the form of a one-page infographic which covered key content, definitions, and examples from each week’s lesson, along with several discussion questions and activities that caregivers could implement in the home with their students. The weekly caregiver nudges were distributed electronically or as a paper flyer to all students receiving the CP-R program.

### Screening Process

Classroom teachers were asked to complete the Teacher Observation of Classroom Adaptation-Checklist (TOCA-C; Bradshaw & Kush, 2019; Koth et al., 2009), a brief teacher-rating tool derived from the earlier TOCA measure (Werthamer-Larsson et al., 1991). The TOCA-C included 33 items for teachers to rate, which were organized into seven established subscales: (1) Concentration Problems, (2) Aggressive/Disruptive Behavior, (3) Prosocial Behavior, (4) Emotion Regulation Problems, (5) Internalizing Problems, (6) Family Problems, and (7) Family Involvement (Bradshaw & Kush, 2019). See additional details below.

The homeroom teacher for each participating classroom was asked to complete this brief (~ 5 min per form) TOCA-C on Qualtrics for each consented student in their class at both pre-test and post-test. Consistent with the TOCA-C administration protocol (Bradshaw & Kush, 2019), homeroom teachers were selected to complete the TOCA-Cs because they had greater familiarity and relational depth with the students than other educators and school staff members. More specifically, homeroom teachers typically serve as the primary providers of students’ academic content, spending more than half of the school-day with their students. Other school staff members, who may have completed the self-report survey, were not asked to complete the TOCA-C behavior rating of individual students. In only one instance did a different individual complete the pre- and post- student behavior ratings, and this was due to an unexpected situation (i.e., the death of the classroom teacher).

Based on the data gathered from the TOCA-C ratings, roughly the 15% of students with the highest TOCA-C scores (on aggressive/ disruptive behavior, emotion regulation problems, and internalizing problems) were invited to participate in the targeted group component of the program which consisted of 12 parallel lessons, as previously described. Thus, the TOCA-C was used both as a screener to identify the student eligible for the targeted group program, as well as to examine the pre-post changes for students on internalizing and externalizing challenges.

### Research Ethics

The project was approved by the Institutional Review Boards at the participating universities (IRB SBS# 2264, IRB-20-0021). The participating school divisions provided approval for all study procedures and data collection activities. Parents provided written informed consent and youth provided assent. School personnel provided written consent. All participation was voluntary.

### Measures

*Teacher Observation of Classroom Adaptation—Checklist* (TOCA-C; Bradshaw & Kush, 2019). As described briefly above, the TOCA-C was completed by the homeroom teacher to capture individual ratings for the students in their classrooms; as noted above, it was also used by the research team as the screening measure to identify children eligible for the targeted group (i.e., Tier 2 or selective) intervention. The TOCA-C was adapted from the original TOCA by Werthamer-Larsson et al. (1991; Bradshaw & Kush, 2019; Koth et al., 2009). Teachers provided individual TOCA-C ratings of students’ classroom behaviors and family engagement levels at two timepoints (pre- and post-intervention). Teachers only provided ratings of students who had parent/guardian consent for participation in the project. When rating each student (on a 6-point Likert scale, 1 = *never* to 6 = *almost always*), teachers were asked to consider the students’ behavior within the past three weeks when responding to items from seven subscales: (1) Concentration Problems, which assesses inattention and off-task behaviors (α = 0.88); (2) Aggressive and Disruptive Behavior, which assesses aggressive, disobedient, and disruptive behaviors (α = 0.88); (3), Prosocial Behavior, which assess social interactions (α = 0.82); (4) Emotion Regulation Problems, which assesses impulsive behaviors as well as a child’s expression of anger or disappointment (α = 0.90), (5) Internalizing Problems, which assesses presentations of low or anxious mood (α = 0.87). (6) Family Problems, which assesses stability of the home environment (α = 0.73), and (7) Family Involvement, which assesses caregiver involvement with school events and offerings (α = 0.90) [all alphas are from the baseline of this study]. Students being rated had been present in class at least 8 days out of the past three weeks. This enhanced version of the TOCA-C was informed by a recent Item Response Theory study, which produced a more concise and focused version of the scale based on teacher ratings of 17,456 children (47.7% female, 54.2% Black, 7.3% Latine). Analysis of differential item functioning revealed no measurement differences by sex, race, or grade level (Bradshaw & Kush, 2019). Teachers also reported some basic demographic characteristics on the TOCA-C, including student sex and grade level.

As noted above, the TOCA-C was completed by the homeroom teacher at both pre-test and post-test; in all but one case, the same homeroom teacher rated the same group of students at pre- and post-intervention; the one exception to this was a teacher who replaced the baseline homeroom teacher who had unexpectedly become unavailable and was no longer at the school. The pre-test TOCA-C collection occurred after teachers had at least one month to learn their students’ behaviors, and post-test data collection approximately 2–4 weeks after the conclusion of the program. On average, 105 days elapsed between pre- and post-test evaluation (range: 77–146 days), with the 12 sessions (roughly 1 per week) occurring in between. Classroom teachers who completed the TOCA-Cs received a modest financial incentive, based upon the number of TOCA-Cs completed, at both pre- and post-implementation.

### Educator Survey

The online educator self-report survey assessed various constructs including Teaching Self-Efficacy, Social Emotional Learning Efficacy, Mindfulness in Teaching, Stress, and Burnout. All survey items followed the same, 0–5 Likert scale of 0 = *Strongly Disagree* to 5 = *Strongly Agree*. Some items were reversed, with higher values indicating more of the construct. This survey was available to all teachers and school staff that interacted with students receiving Coping Power on a regular basis, including those staff that did not implement the program or complete the TOCA-C student behavior ratings [all alphas are from the baseline of this study].

*Teacher-Reported Teaching Self-Efficacy* Five items were administered from the Personal Teaching Efficacy scale (Hoy & Woolfolk, 1993; e.g., “I can manage almost any student behavior problem”; α = 0.90). This measure focused on teachers’ belief in their ability to respond to challenging student behavior.

*Teacher-Reported Occupational Burnout* Teachers completed a brief, 4-item assessment of burnout using a subset of items from the emotional exhaustion subscale of the Burnout Inventory by Maslach and Jackson (1981). Sample items included “I feel like I am at the end of my rope” (α = 0.87). Teachers reported the frequency with which they experienced job-related stressors. Higher scores reflected more emotional exhaustion.

*Teacher-Reported Stress* Participants completed five items from the Exposure to Job Stress measure (Hurrell & McLaney, 1988) to assess their work-related stress (e.g., “I am unable to cope with the stress of my job on a daily basis”; α = 0.78). Higher scores reflected more stress.

*Social Emotional Learning Efficacy* Eight items were administered from the Social Emotional Learning Efficacy scale (Domitrovich et al., 2008); “I am able to help children develop self-control”; α = .92). This measure focused on teachers’ beliefs in their ability to understand and respond to the social and emotional needs of students in the classroom.

*Mindfulness in Teaching* Participants completed 14 items from the Mindfulness in Teaching measure (Frank et al.,^2016^) to assess their level of mindfulness while teaching (e.g., “I am aware of how my moods affect the way I treat my students”; α = 0.84). Higher scores reflected greater rates of mindfulness.

*Teacher Demographics* As a part of the teacher self-report survey, teachers reported on their demographic information including their sex (male = 1, female = 0), race/ethnicity (Black/African American, White, Multiracial or other), and grade taught (4th -7th or did not teach). Any teachers of students receiving CP-R were eligible to complete the educator survey, including special education teachers, ELL teachers, paraprofessionals, and specials teachers.

All school employees (e.g., teachers, counselors) who had regular interaction with the participating 4th -7th grade students (e.g., special education teachers, librarians, gym teachers, etc.) were asked to complete this self-report survey to explore the potential for changes in the school climate, self-efficacy, and overall wellness factors. We asked non-implementing educators to complete this survey to explore the potential for “spill-over” effects of the program, as all these educators had received the electronic nudges and access to the website resources, and thus were familiar with the core elements of the program and tips for implementing strategies. The participating educators and school staff completed the measure online (pre- and post-implementation data collection periods); they received a $25 gift card for completing each of the two waves of data.

#### Coping Power-Rural Implementation Measures

*Implementation Log* Implementation logs were completed by school-based CP-R implementers following the completion of each lesson (i.e., classroom or targeted group). These logs captured the degree of completion across four lesson components (i.e., warm-up and previous lesson review, skill introduction activity, skill practice activity, and summary and wrap-up). Additional data were collected regarding student comfort with peers and implementers, as well as descriptive data (e.g., number of students present, length of lesson, preparation time).

*Coaching Log* Coaches completed logs after each coaching session with program implementers. Coaches indicated completion of five coaching components: initial engagement and rapport building, review of program successes and challenges, plans for future implementation, discussion of material usage (e.g., caregiver and teacher nudges, workbooks), as well as time to wrap-up and reflect.

### Analyses

All student participants enrolled in the pilot study received the universal Coping Power-*Rural* intervention, so we estimated the promise of the program by comparing pre-test and post-test assessments of our outcomes. Statistically, we examined this by assessing the effect of time on outcomes while using cluster-robust standard errors (McNeish et al., 2017) to account for within-participant dependence, allowing us to estimate our effects in single-level models. Models predicting student outcomes were run at the student level while models predicting teacher outcomes were run at the teacher level. A dummy-coded indicator of whether the participant was in a middle school was included in both student and teacher models. Coefficients for all predictive models were obtained in using full information maximum likelihood (FIML) estimation in M*plus* version 8.8 (Muthén & Muthén, 1998–2022). FIML accounts for missingness by using all available data from each case to estimate parameters and adjust for potential bias in the estimates resulting from missing data. This approach provides unbiased estimates in the presence of data missing at random and has been identified as one of the optimal ways to handle missing data (Peugh & Enders, 2004). Due to the lack of a control group, we examined pre-post differences, focused on the TOCA-C teacher-reported assessments of student behavior and an educator survey comprising various self-reported measures of teaching efficacy and wellness. Each instrument was analyzed using a separate model, with results presented individually below.

## Results

### Implementation Findings

The implementation findings are summarized in Table 2. This includes contacts between coaches and teachers recorded in the coaching log (see Table 2A). Importantly, all implementers received the three required coaching sessions, but some implementers requested the additional optional coaching sessions or were more interactive with the coach over email. Table 2B reports the Coping Power-*Rural* implementation fidelity across three time points during the intervention. Finally, Table 2C summarizes the implementation of CP-R in both the universal classroom and targeted group. On average, implementers reported that each universal classroom lessons took an average of 36.25 min (*SD* = 9.04) to complete and an average of 32.90 min (*SD* = 17.12) to prepare for. The preparation and implementation time for each targeted group session was slightly shorter at 23.55 (*SD* = 12.91) minutes for preparation and 33.87 min (*SD* = 6.25) for implementation.

### Pre-post Comparisons for Student Outcomes

To examine our first aim regarding changes in student outcomes, we present the pre-test, post-test, and change score estimates for each TOCA-C subscale, along with a test of whether the pre-test-to-post-test change was significantly different from zero in Table 3. The ratings of conduct problems, aggressive/disruptive behavior, emotion regulation problems, internalizing problems, and family problems significantly improved from pre-test to post-test.

**Table 3:** Pre-test, post-test, and change estimates

TOCA-C subscale	Pre-test		Post-test		Post-test-pre-test change				
	Estimate	*SE*	Estimate	*SE*	Estimate	*SE*	*p*	Cohen’s d	Hedges’ g
Conduct problems	1.997	0.106	1.781	0.103	−0.216	0.098	0.028	−0.162	−0.161
Aggressive/disruptive behavior	1.927	0.087	1.735	0.065	−0.192	0.071	0.007	−0.199	−0.198
Prosocial behavior	4.578	0.086	4.668	0.084	0.090	0.078	0.247	0.085	0.084
Emotion regulation problems	2.372	0.112	2.056	0.085	−0.315	0.095	0.001	−0.244	−0.243
Internalizing problems	1.940	0.086	1.488	0.058	−0.452	0.068	< 0.001	−0.489	−0.487
Family problems	1.443	0.105	1.058	0.085	−0.385	0.095	< 0.001	−0.298	−0.297
Family involvement	5.113	0.097	5.254	0.089	0.140	0.094	0.137	0.110	0.109

Educator self-report subscales	Pre-test		Post-test		Post-test-pre-test change				
	Estimate	*SE*	Estimate	*SE*	Estimate	*SE*	*p*	Cohen’s d	Hedges’ g
*Educator self-report survey pre-test, post-test, and change estimates*									
Teaching efficacy	4.555	0.117	4.994	0.114	0.439	0.125	< 0.001	0.785	0.755
Social-emotional Learning efficacy	4.678	0.087	5.030	0.093	0.352	0.099	< 0.001	0.795	0.765
Mindfulness in teaching	4.709	0.093	4.717	0.107	0.007	0.120	0.950	0.013	0.013
Teacher stress	3.329	0.155	3.105	0.145	−0.224	0.140	0.111	−0.358	−0.344
Burnout	2.926	0.151	2.710	0.159	−0.216	0.150	0.150	−0.322	−0.310

*TOCA-C* teacher observation of classroom adaptation-checklist. All estimates control for a middle school indicator coded 0 = Not a middle school, 1 = Is a middle school

### Moderation of Student Outcomes by Targeted Group Participation

For Aim 2, we explored whether these effects were moderated by student sex or participation in the targeted group intervention. Given that these were student-level moderators, we only examined their effects on the TOCA-C, which comprised student-level outcomes. Student sex did not influence the pre-post difference for any of our outcomes (all *p*’s > .10). Tests of the intervention by participation in the targeted group interaction are presented in Table 4, while individual pre-test and post-test means by targeted group status are presented in Supplemental Material B. We observed that students in the targeted group experienced significantly more positive pre-post changes on aggressive/disruptive behaviors, emotion regulation problems, and internalizing problems compared to those exposed to just the universal program. However, participation in the targeted group was not associated with pre-post changes on conduct problems, prosocial behavior, family problems, or family involvement.

**Table 4:** Universal vs. targeted group participation interaction effects on the teacher observation of classroom adaptation-checklist (TOCA-C)

TOCA-C subscale	Universal only		Targeted group		Targeted group vs. universal only difference				
	Intervention effect	*SE*	Intervention effect	*SE*	Estimate	*SE*	*p*	Cohen’s d	Hedges’ g
Conduct problems	−0.135	0.105	−0.625	0.233	−0.491	0.253	0.053	−0.143	−0.142
Aggressive/disruptive behavior	−0.124	0.0703	−0.547	0.184	−0.423	0.194	0.029	−0.160	−0.160
Prosocial behavior	0.098	0.085	0.106	0.181	0.009	0.199	0.965	0.003	0.003
Emotion regulation problems	−0.193	0.095	−0.940	0.213	−0.747	0.228	0.001	−0.241	−0.240
Internalizing problems	−0.377	0.067	−0.818	0.195	−0.441	0.205	0.032	−0.158	−0.158
Family problems	−0.347	0.101	−0.611	0.239	−0.264	0.259	0.307	−0.075	−0.075
Family involvement	0.124	0.096	0.256	0.254	0.132	0.268	0.622	0.036	0.036

*TOCA-C* teacher observation of classroom adaptation-checklist. All estimates control for a middle school indicator coded 0 = Not a middle school, 1 = Is a middle school

### Pre-post Comparisons for Educator Self-Reported Outcomes

To explore Aim 3, we examined pre-test, post-test, and change score estimates for each educator survey scale, along with a test of whether the pre-test-to-post-test change was significantly different from zero (see Table 3). We found that teaching efficacy and social-emotional learning efficacy significantly increased from pre-test to post-test. There were no significant pre-post changes in mindfulness in teaching, teacher stress, or burnout.

### Sensitivity Analyses

We conducted a series of sensitivity analyses to explore if the pre-test vs. post-test differences reported above varied as a function of whether the educators participated in the initial CP-R training or whether they had been selected to provide student ratings on the TOCA-C. Given that the CP-R program is designed to have broad effects across all adults who had contact with the participating students (as designed through the distribution of electronic nudges and access to the web-based resources), we did not expect to find differences based on these sensitivity analyses when accounting for training status and completion of the TOCA-C ratings. First, we estimated a model exploring gains in educator self-report ratings after controlling for both initial training status and TOCA-C rater status and found that including these covariates did not substantially influence results (see Supplemental Material C). We then estimated a model examining whether either of these factors moderated the pre-test to post-test gains, but we found no significant effects (see Supplemental Material D). In combination, these sensitivity analyses suggested that the pre-post changes did not vary significantly when accounting for initial training status or TOCA-C rater status.

## Discussion

This pilot study aimed to examine the preliminary pre-post changes associated with implementation of the Coping Power*-Rural* program within a single school year. Although the original Coping Power program was largely used as a targeted group intervention focused on reducing aggressive and disruptive behaviors for youth who had screened into the program, Coping Power*-Rural* used a two-tiered approach, incorporating both a universal classroom-wide and targeted group component. It also leveraged cognitive behavioral strategies to target emotion regulation, social problem-solving, and caregiver engagement. Therefore, this adapted version aimed to extend the program beyond a single diagnostic category to address a range of transdiagnostic concerns (e.g., anxiety, depression) (Clifford et al., 2020; Hersh et al., 2016).

These findings indicated significant pre-post changes on the TOCA-C across all subscales. Specifically, there were pre-post improvements in conduct problems, aggressive/disruptive behavior, prosocial behavior, emotion regulation problems, internalizing problems, as well as family problems and family involvement. The effect sizes were in the small to moderate range (0.17–0.48). One particularly promising finding is the transdiagnostic effect for the internalizing symptoms, which had the largest effect size change (Hedge’s g = 0.48). CP-R was designed with this goal in mind (Nguyen et al., 2024), as rural schools and communities may have limited access to specialized mental health providers, which often presents a barrier to diagnostic assessment and treatment (Andrilla et al., 2018). Although relatively small, and tentative due to the non-experimental design and lack of follow-up data, these pilot findings are promising, particularly given the relatively short time period for delivery.

Similarly, significant improvements for students in the targeted group program relative to those students who were only exposed to the universal classroom programming were noted. Specifically, students participating in the targeted group showed greater reductions in teacher ratings of conduct problems, aggressive/disruptive behaviors, emotion regulation, and internalizing problems. Although the Hedge’s gs were smaller, ranging from 0.14 to 0.24, for these results, they still suggest a promising added benefit of the targeted group. It appears that students who participated in the targeted group intervention exhibited additional reductions in multiple problem areas, thereby suggesting that participation in the more intensive targeted group sessions yield additional benefits for these higher-risk youth, including some transdiagnostic improvements in internalizing symptoms (Clifford et al., 2020; McDaniel et al., 2025).

Finally, for the educator self-report data, we saw significant pre-post changes in their teaching efficacy and social emotional-learning efficacy (Hedge’s gs were 0.48 and 50, respectively). This is promising, given many of the educators did not participate in the formal training and were not the actual implementers of the program, but rather received the electronic nudge and website materials, and may have witnessed the counselor implementing the program. Our sensitivity analyses indicated that exposure to the initial training did not have an added benefit on perceived teaching self-efficacy more generally, or efficacy related to implementing social-emotional learning content. These findings echo some similar results from work by Domitrovich and colleagues (2016), which found positive impacts on teachers who delivered a combined classroom management program combined with social-emotional learning content (i.e., Good Behavior Game combined with the Promoting Alternative Thinking Strategies Program). Taken together, these findings suggest that delivery of the program content electronically through the nudges and/or website may hold promise for influencing educator experiences, regardless of direct exposure to CP-R through the initial training or program delivery.

### Limitations and Future Directions

This pilot study has some limitations, such as our focus on an intervention condition only and a pre-post design, which precludes causal inference. A more rigorous research design, which includes randomization, would provide stronger evidence of impact. Although the pre–post design cannot fully rule out the influence of historical confounds, it can provide us with a preliminary look at the effects of the program. Moreover, the within-participants design controls for stable individual differences, reducing the risk of confounds arising from between-participant variability. Future research should also explore long-term outcomes, particularly the extent to which behavioral improvements are maintained beyond the intervention period over time. Additionally, future studies should include observations and/or student reports, to reduce reliance on staff reports.

As noted above, although we provided the schools with the option of having teachers or clinicians implement the program, most schools opted to have the program delivered by the counselors, while the teachers observed; having teachers deliver the classroom programming may yield a strong impact on both students and teachers (Domitrovich et al., 2016). The mechanism by which the “spill-over” effects occurred for non-implementing educators is unclear, however, we hypothesize it may have been through their receipt of the electronic program content. Further research is needed to better understand the mechanism of change for these educators, relative to those who had direct exposure to the program or experience delivering the program (see Domitrovich et al., 2016). Caregiver receipt of the parenting materials was unknown and may vary by school or mode of delivery of the program content. Although implementers did send content home via email or physical flyer, we did not have a method to track caregiver review of or engagement with the materials in the current pilot study.

In conclusion, this study adds to the evidence base for Coping Power, suggesting the CP-R adaptation is potentially promising for rural upper elementary and middle school students. As noted, rural communities may have limited access to resources for mental health and behavioral intervention. By reducing the need for multiple interventions targeting various mental health and behavioral concerns by using a single multicomponent program to address universal and more targeted needs of youth, CP-R presents a pragmatic approach to improve youth mental health in rural communities. As rural schools continue to serve as the primary access point for mental health services for youth, Coping Power-*Rural* appears to be a potentially promising approach for reducing behavioral and mental health risks in these settings.

## Electronic Supplementary Material

Below is the link to the electronic supplementary material.

Supplementary Material 1
