# Supplementary material for: Coping Power-Rural: A Pilot Study of the Two-Tiered Preventive Intervention Focused on Addressing Transdiagnostic Needs in Rural Schools
Source: School Ment Health. 2026 Apr 21;18(3):808–21. doi: 10.1007/s12310-026-09873-3 (PMC13476284; doi:10.1007/s12310-026-09873-3)
Supplement: Supplementary file 1 [file 12310_2026_9873_MOESM1_ESM.docx]

# **Supplemental Material A.** *Educator Demographics by Timepoint and Subgroups.*

|  | Count [*N*] (Percent) | | | | | | | |
| --- | --- | --- | --- | --- | --- | --- | --- | --- |
|  | Pre-test | | | | Post-test | | | |
| Participant  Characteristic | No Training | Attended Training | Did Not  Give Ratings | Gave Ratings | No Training | Attended Training | Did Not  Give Ratings | Gave Ratings |
| Grade Level |  |  |  |  |  |  |  |  |
| 4th Grade | 8 (25.0%) | - | 1 (3.6%) | 7 (35.0%) | 8 (22.9%) | - | 1 (3.4%) | 7 (35.0%) |
| 5th Grade | 7 (21.9%) | - | - | 7 (35.0%) | 8 (22.9%) | - | 1 (3.4%) | 7 (35.0%) |
| 6th Grade | - | 2 (12.5%) | - | 2 (10.0%) | - | 2 (14.3%) | - | 2 (10.0%) |
| 7th Grade | 1 (3.1%) | 4 (25.0%) | 1 (3.6%) | 4 (20.0%) | 1 (2.9%) | 3 (21.4%) | 1 (3.4%) | 3 (15.0% |
| 8th Grade | 1 (3.1%) | - | 1 (3.6%) | - | 1 (2.9%) | - | 1 (3.4%) | 1 (5.0%) |
| Multiple Grades | 11 (34.4%) | 4 (25.0%) | 15 (53.6%) | - | 13 (37.1%) | 7 (50%) | 19 (65.5%) | - |
| Did not Teach | 4 (12.5%) | 6 (37.5%) | 10 (35.7%) | - | 4 (11.4%) | 2 (14.3%) | 6 (20.7%) | - |
| Race/Ethnicity |  |  |  |  |  |  |  |  |
| Black/African American | 1 (3.1%) | 8 (50.0%) | 3 (10.7%) | 6 (30.0%) | - | 8 (7.1%) | 2 (6.9%) | 6 (30.0%) |
| Hispanic/Latine | - | - | - | - | 1 (2.9%) | - | 1 (3.4%) | - |
| White | 31 (96.9%) | 8 (50.0%) | 25 (89.3%) | 14 (70.0%) | 34 (97.1%) | 5 (35.7%) | 25 (86.2%) | 14 (70.0%) |
| Multiracial or Other | - | - | - | - | - | 1 (7.1%) | 1 (3.4%) | - |
| Sex |  |  |  |  |  |  |  |  |
| Female | 30 (93.8%) | 15 (93.8%) | 27 (96.4%) | 18 (90.0%) | 33 (94.3%) | 12 (85.7%) | 27 (93.1%) | 18 (90.0%) |
| Male | 2 (6.3%) | 1 (6.3%) | 1 (3.6%) | 2 (10.0%) | 2 (5.7%) | 2 (14.3%) | 2 (6.9%) | 2 (10.0%) |

# **Supplemental Material B.** *Means and Standard Errors by Timepoint and Targeted Group Participation on the Teacher Observation of Classroom Adaptation-Checklist (TOCA-C).*

|  | Universal Only | | | |  | Targeted Group | | | |  |
| --- | --- | --- | --- | --- | --- | --- | --- | --- | --- | --- |
| TOCA-C Subscale | Pretest | | Posttest | |  | Pretest | | Posttest | |  |
|  | Mean | *SE* | Mean | *SE* |  | Mean | *SE* | Mean | *SE* |  |
| Conduct Problems | 1.851 | .111 | 1.716 | .117 |  | 2.699 | .187 | 2.074 | .171 |  |
| Aggressive/  Disruptive Behavior | 1.778 | .086 | 1.654 | .069 |  | 2.648 | .211 | 2.100 | .161 |  |
| Prosocial Behavior | 4.671 | .087 | 4.769 | .090 |  | 4.118 | .181 | 4.225 | .169 |  |
| Emotion Regulation Problems | 2.134 | .106 | 1.941 | .093 |  | 3.516 | .210 | 2.576 | .166 |  |
| Internalizing Problems | 1.833 | .080 | 1.457 | .057 |  | 2.452 | .228 | 1.634 | .154 |  |
| Family Problems | 1.305 | .102 | .958 | .082 |  | 2.118 | .254 | 1.507 | .226 |  |
| Family Involvement | 5.212 | .094 | 5.336 | .091 |  | 4.633 | .262 | 4.890 | .242 |  |

# **Supplemental Material C.** *Unstandardized Coefficients from Models Including Training Status and Rating Status as Covariates.*

|  | b (SE),  *p* | | | | |
| --- | --- | --- | --- | --- | --- |
|  | Teaching Efficacy | Social-Emotional Learning Efficacy | Mindfulness in Teaching | Teacher Stress | Burnout |
| Intercept | 4.309 (.153),  *p* < .001 | 4.510 (.115),  *p* < .001 | 4.652 (.139),  *p* < .001 | 3.347 (.219),  *p* < .001 | 3.107 (.221),  *p* < .001 |
| Timepoint indicator | .444 (.118),  *p* < .001 | .357 (.094),  *p* < .001 | .009 (.120),  *p* = .939 | -.233 (.138),  *p* = .092 | -.223 (.149),  *p* = .133 |
| Training indicator | .479 (.207),  *p* = .021 | .372 (.195),  *p* = .057 | .175 (.193),  *p* = .365 | -.465 (.289),  *p* = .108 | -.425 (.290),  *p* = .143 |
| Rating status indicator | .319 (.190),  *p* = .092 | .183 (.162),  *p* = .259 | .028 (.161),  *p* = .862 | .299 (.254),  *p* = .240 | .067 (.291),  *p* = .816 |
| Middle school indicator | -1.069 (.482),  *p* = .027 | -.601 (.340),  *p* = .077 | .056 (.369),  *p* = .879 | -.194 (.352),  *p* = .580 | -.278 (.503),  *p* = .580 |

Note: Timepoint indicator coded 0 = pretest, 1 = posttest; Training indicator coded 0 = No training, 1 = Received training; Rating status indicator coded 0 = Did not give ratings, 1 = gave ratings; Middle school indicator coded 0 = Not a middle school, 1 = Is a middle school. The coefficient for the timepoint indicator estimates posttest-pretest gains, while the p-value for that coefficient indicates whether gains are significantly different from zero.

# **Supplemental Material D.** *Unstandardized Coefficients from Models Testing Training Status and Rating Status Moderation Effects On Educator Self-Reported Measures.*

|  | b (SE),  *p* | | | | |
| --- | --- | --- | --- | --- | --- |
|  | Teaching Efficacy | Social-Emotional Learning Efficacy | Mindfulness in Teaching | Teacher Stress | Burnout |
| Intercept | 4.279 (.169),  *p* < .001 | 4.505 (.138),  *p* < .001 | 4.570 (.141),  *p* < .001 | 3.372 (.250),  *p* < .001 | 3.062 (.235),  *p* < .001 |
| Timepoint indicator | .498 (.185),  *p* = .007 | .363 (.160),  *p* = .023 | .166 (.159),  *p* = .296 | -.281 (.229),  *p* = .219 | -.307 (.207),  *p* = .138 |
| Training indicator | .660 (.240),  *p* = .006 | .486 (.224),  *p* = .030 | .177 (.234),  *p* = .450 | -.482 (.345),  *p* = .163 | -.548 (.322),  *p* = .089 |
| Rating status indicator | .249 (.226),  *p* = .270 | .104 (.195),  *p* = .592 | .223 (.173),  *p* = .196 | .250 (.298),  *p* = .402 | .055 (.321),  *p* = .863 |
| Middle school indicator | -1.078 (.480),  *p* = .025 | -.602 (.338),  *p* = .075 | .033 (.364),  *p* = .929 | -.186 (.351),  *p* = .597 | -.263 (.509),  *p* = .605 |
| Timepoint × Training | -.382 (.249),  *p* = .125 | -.245 (.187),  *p* = .191 | .018 (.281),  *p* = .249 | .025 (.314),  *p* = .936 | .250 (.344),  *p* = .467 |
| Timepoint × Rating | .162 (.231),  *p* = .483 | .171 (.189),  *p* = .364 | -.387 (.242),  *p* = .110 | .096 (.270),  *p* = .722 | .010 (.299),  *p* = .974 |

Note: Timepoint indicator coded 0 = pretest, 1 = posttest; Training indicator coded 0 = No training, 1 = Received training; Rating status indicator coded 0 = Did not provided TOCA-C ratings, 1 = Provided TOCA-C ratings; Middle school indicator coded 0 = Not a middle school, 1 = Is a middle school.
